# Cytogenetic and Clinical Assessment of a Family with Treacher Collins Syndrome

**DOI:** 10.1155/2011/708450

**Published:** 2011-06-23

**Authors:** Manoj Kumar, Rakesh Kumar, Mukesh Tanwar, Supriyo Ghose, Jasbir Kaur, Rima Dada

**Affiliations:** ^1^Laboratory for Molecular Reproduction and Genetics, Department of Anatomy, All India Institute of Medical Sciences, New Delhi 110029, India; ^2^Dr. R.P. Centre for Ophthalmic Sciences, All India Institute of Medical Sciences, New Delhi 110029, India; ^3^Department of Ocular Biochemistry, Dr. R.P. Centre for Ophthalmic Sciences, All India Institute of Medical Sciences, New Delhi 110029, India

## Abstract

Treacher Collins syndrome (TCS) is a rare autosomal dominant disorder characterized by craniofacial deformities. It is the most common type of mandibulofacial dysostosis (MFD). The objective of this study is to do cytogenetic analysis of a TCS family. Physical examination and all available medical records were reviewed. 50 GTG-banded metaphases were analysed to detect any structural or numerical chromosomal abnormality. Downward slanting of palpebral fissures, hypoplasia of zygomatic arch complex, and hypoplasia of mandible were present in all. Cytogenetic findings show interstitial deletion in chromosomes 5(q32-q33) and 3(q23–q25). We report four members of three generations of a family having TCS in a unique way that the deletion has been found in 3q and 5q which has not been reported. Mosaicism of deletion on 5q was detected in all affected members whereas 3q deletion was found only in one member (II.2). This finding may represent a more severe manifestation of the TCS. Thus the evaluation and counselling of the TCS patients should be undertaken with caution.

## 1. Introduction

TCS is a rare genetic disorder characterised by craniofacial deformities. It is named after Edward Treacher Collins (1862–1932), the English surgeon and ophthalmologist who described its essential traits in 1900. TCS is an autosomal dominant disorder which has an incidence of approximately 1 in 50,000 still births [1]. This is a congenital malformation involving dysmorphogenesis of first and second branchial arches which occurs between the 5th to 8th week of embryonic development [2]. There is no preference among the genders or races. The clinical features of the TCS are usually bilaterally symmetrical in nature. Mildly affected individuals are difficult to diagnose because of the variable expressivity of the gene; however, the gene is rarely nonpenetrant [3]. But the major diagnostic criteria of this disease include antimongoloid slant of the palpebral fissures, malar hypoplasia, malformation of the pinna, mandibular hypoplasia, coloboma of the eyelid, micrognathia, microtia, conductive deafness, and cleft palate [4, 5]. In severely affected patients the airway is compromised by the mandibular deficiency, glossoptosis, and choanal atresia [6, 7]. 

The locus of TCS was initially mapped to a 9cM region on chromosome 5q31–34 [3]. Subsequently, a fine genetic and radiation hybrid mapping helped in establishing a critical region of <1 Mb at 5q31.3–32 to be identified as TCS locus [8–10]. The region contains the TCOF1 gene which codes for a low-complexity protein, treacle composed of 1411 amino acids. This protein plays a fundamental role in early embryonic development, particularly in the development of craniofacial complex. The peak levels of expression of the protein in the developing embryos were observed at the edge of neural folds and in the branchial arches at the time of critical morphogenetic events [11]. Treacle is considered as the nucleolar localization signal binding protein that travels on the track between cytoplasm and the nucleolus [12]. Mutations are spread throughout the gene, and 60% of the TCS cases arise from de novo mutations [13]. The patients with TCS were found to be heterozygous for mutation in TCOF1 gene [14, 15].

Cytogenetic abnormalities have been useful in directing attention to candidate region in craniofacial anomalies in which the biochemical defect is not known. Chromosomal location in these cases has also been proven successfully through genetic linkage analysis [13, 16–19]. There are several reports describing the patients with various chromosomal abnormalities suggesting additional loci for TCS on chromosomes 3p23–24.12, 4p15.32–14, and 5q11 [20–22]. Thus the possibility exists that TCS can be caused by more than one gene because recombination in affected individuals has been reported to preclude TCOF1 as the disease causing gene located on 5q32-33 [23]. 

We report a family in which four members of three generations are affected with TCS. The chromosomal analysis of the affected family members was done using GTG-banding which revealed a unique concordance of 3q and 5q deletions, never reported earlier. Although the clinical features of all the affected members are similar but a new deletion has been found in one member. This suggests that TCS locus might be positioned at a locus other than 5q32-33 but the chromosomal analysis of the other three members excluded this possibility.

## 2. Case Presentation

### 2.1. Clinical Features

**Figure 1:**
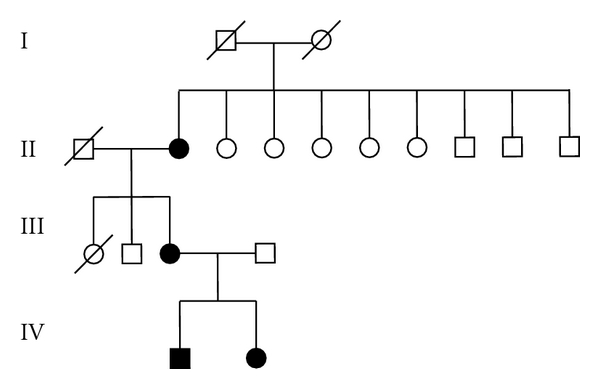
Pedigree showing the autosomal dominant pattern of inheritance of TCS in the family.

**Figure 2:**
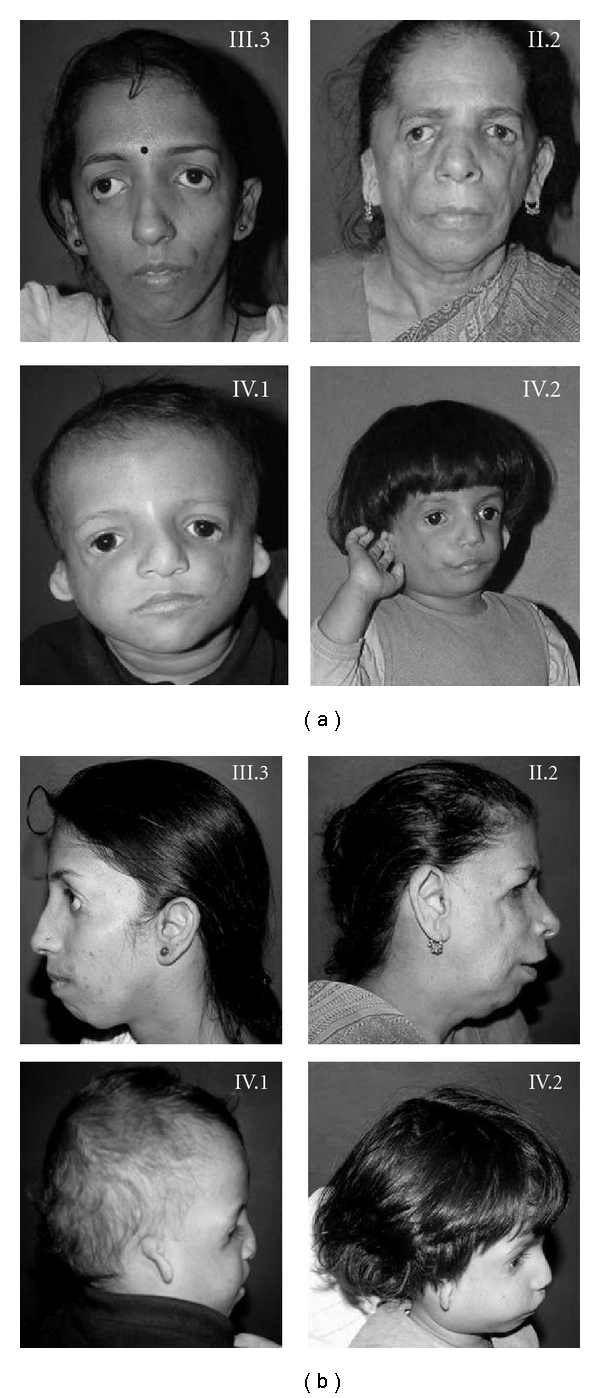
(a) Showing the phenotypes of the effected members (front view). (b) Showing the phenotypes of the effected members (side view).

All affected members had normal psychomotor development and intelligence. Downward slanting palpebral fissures, hypoplasia of mandible, and hypoplasia of zygomatic arches are the only clinical features present in each affected member whereas three out of four show partial absence of lower eyelashes. None of the affected members had microphthalmia, epibulbar lipodermoid, upper or lower lid coloboma, hair on the cheeks, microstomia, and choanal atresia. Pedigree (Figure 1) showed the transmission of the trait as autosomal dominant pattern. It showed two female to female and one female to male transmission. There is no significant difference in clinical severity between the affected males and females. Phenotypic features of the affected members have been summarized in Table 1, and the photographs of the affected members have been shown in Figures 2(a) and 2(b).

### 2.2. Cytogenetic Evaluation

#### 2.2.1. Chromosome Preparation [24]

Chromosomal analysis was done in TCS patients to identify for the presence of any numerical or structural chromosomal aberrations. For this lymphocyte cultures were setup, and chromosomes were analyzed by G-banding. Five mL of heparinized blood was drawn and kept in an upright position at 37°C for 30 minutes. This helps in the separation of plasma from red blood cells. Then, the plasma and the settled lymphocyte (PLS, plasma lymphocyte suspension) in buffy coat tapped gently and mixed together. The needle was bent, and 0.5 mL of PLS was transferred into a sterile culture vial containing 5 mL of media RPMI-1640 and 0.2 mL Phytohaemagglutinin (PHA). The cultures were incubated for 72 hours at 37°C. After 70 hours of incubation, 0.1 mL (0.2%) of colcemid was added to the cultures. At 72 hours the samples were washed for removing colcemid. Then, they were centrifuged at a speed of 1000 rpm for 10 minutes, the supernatant was discarded, and freshly prepared pre-warmed hypotonic solution (0.56% KCl) was added and incubated for 20–25 minutes at 37°C. The cell suspension centrifuged again, and after discarding the supernatant, freshly prepared chilled carnoy's fixative (methanol : acetic acid/3 : 1) was added to the cell pellet slowly. At least three changes of fixative were given till the pellet became pale. Two drops of cell suspension were dropped from a height on a clean wet slide.

#### 2.2.2. G-Banding [25]

Giemsa staining of chromosome preparation after proteolytic enzyme treatment revealed G-banding. The 3-day old matured unstained chromosome preparations were flooded with 0.25% trypsin for 10–15 seconds, then the slides were rinsed in phosphate buffer saline. The slides were stained in 2% Giemsa stain for 5–7 minutes, thereafter, they were washed in distilled water. Metaphases were analyzed using cytovision software (zeiss microscope) classified according to ISCN 1995. At least 50 metaphases in each patient were analyzed and karyotyped. 

**Figure 3:**
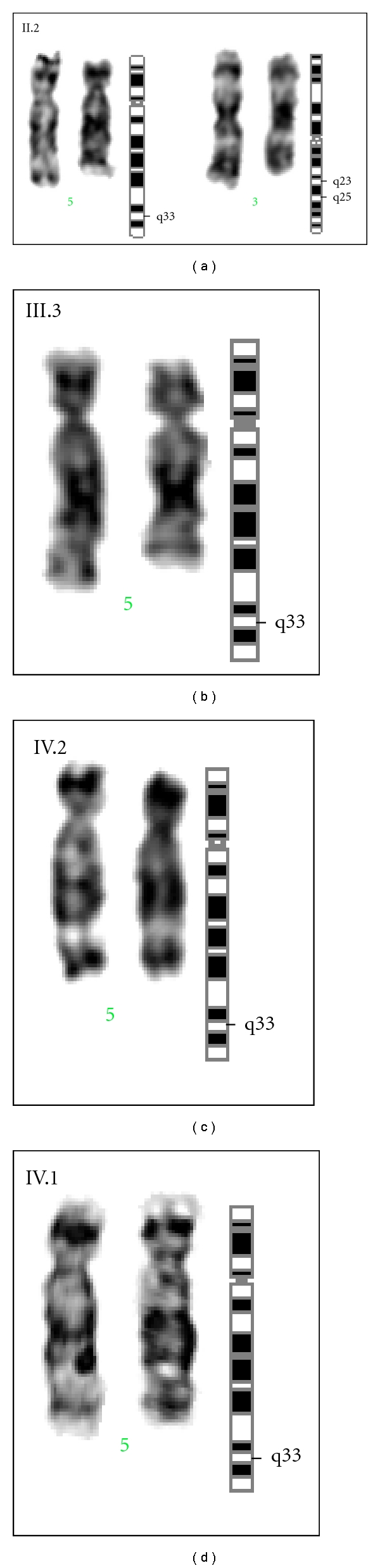
(a) G-banded image of chromosome 3 and 5 showing del(5)(q32-33)del(3q)(q23–25) in II.2. (b) G-banded image of chromosome 5 showing del(5)(q32-33) III.3. (c) G-banded image of chromosome 5 showing del(5)(q32-33) IV.2. (d) G-banded image of chromosome 5 showing del(5)(q32-33) in IV.1.

Cytogenetic studies of peripheral blood lymphocytes showed mosaicism with an interstitial deletion of 3q and 5q. Using 400–450 band level GTG banding, the deleted portion was found to be at 3q23–25 and 5q32-33. Karyotype of the proband (IV.1) (Figure 3(d)) is 46,XY,del(5)(q32-33). The karyotype of his mother (III.3) (Figure 3(a)) and sister (IV.2) (Figure 3(c)) also showed deletion in chromosome 5 only. The chromosomal complement of the grandmother (II.2) (Figure 3(b)) showed deletion in both 3q and 5q, karyotype 46,XX,del(5)(q32-33)del(3q)(q23–25). Mosaicism of 3q and 5q deletion in affected members has been summarized in Table 2. 

## 3. Discussion

TCS is the most common type of mandibulofacial dystosis. This study shows three generations of a family being affected by TCS. Due to variable expressivity of the gene responsible for the TCS, the phenotypic expression of the affected individuals and obligate carriers varies. Thus the diagnosis in obligate carriers is difficult. In our study the affected members can be easily diagnosed as they all have downward slanting palpebral fissures, hypoplasia of mandible, and hypoplasia of zygomatic arches. Thus the affected members have typical clinical features of TCS. The mode of inheritance in the pedigree is consistent with autosomal dominant (AD) pattern of inheritance. TCS is the most common type of AD mandibulofacial dysostosis. Various reports show a patient with TCS and a de novo deletion of region 4p15.32–p14 [26] a girl with a de novo balanced translocation involving chromosome 5 and 13, t(5;13)(q11;p11) [21] and an association of TCS and a balanced translocation t(6;16)(p21.31;p13.11) [18], although unaffected family members also were found to carry translocation. The existence of a similar phenotype in affected individual with detected chromosomal rearrangements supports the existence of genetic heterogeneity of TCS/MFD. However, these chromosomal regions may not be the candidate regions for TCS. It has been shown in a family with MFD and an apparently balanced translocation t(6;16)(p21.31;p13.11); another child with MFD had a normal karyotype, and this translocation did not segregate with this disease. 

To the best of our knowledge, there is no report in the literature, which shows the concordance between TCS and 3q2 deletion. Cytogenetic findings in our case show the deletion in 5q3 and 3q2 within a family which has never been reported earlier. Deletion in chromosome 5 has been detected in all the affected members whereas deletion in chromosome 3 has been detected only in one member of the family. We initially thought that our findings may possibly support the genetic heterogeneity, with the 3q2 deletion causing TCS. However, the detection of 5q3 deletion does not support this conclusion. One member of the family (II.2), despite having 3q2 deletion, had 5q3 deletion that excluded the 3q2 being the candidate region for TCS. The deleted 3q23–25 region has been mapped for BPES, primary ovarian failure (POF3), Bruck syndrome 2, autism (AUTS8), short stature (SHOX2), Asperger syndrome, and Leukemia (AML, MLF1). But review of several reported cases and some of obligatory features of BPES concluded that it has been linked to 3q23–25 region in our case. The region contains a forkhead transcription factor (FOXL2) gene, mutation in which causes BPES. However BPES also has been found in patients showing 7q deletion which shows that BPES is also of genetically heterogeneous entity and it may result from contiguous gene defect [27, 28]. BPES may be with premature ovarian failure (type I) or without premature ovarian failure (type II). Blepharophimosis and ptosis are the most common signs of the BPES whereas less frequently observed anomalies are telecanthus, microphthalmia, broad nasal bridge, strabismus, ventricular septal defect, convex arches of the eyebrows and abnormalities of finger and toes or foot deformities. Micrognathia, cleft palate, hearing loss, and malformed ears are some of the phenotypes which are common in both TCS and BPES. In our case none of the specific features of BPES were present in the affected members. The karyotype of affected members shows mosaicism, and the 3q deletion has been found to be 10% (II.2). It shows that the number of cells with 3q deletion is very low which could be another reason for absence of BPES phenotypes. 

In summary we propose that we found a novel deletion at 3q23–25 in this family. The phenotype is consistent with that of TCS. Screening and detailed genetic analysis of more patients of TCS should be done to study the alternative candidate loci for TCS.

## Figures and Tables

**Table 1:** Phenotypic features of the affected family members.

Affected members	II.2	III.3	IV.2	IV.1
Sex/age	F/55	F/30	F/4	M/3
Downward slanting palpebral fissures	+	+	+	+
Lower lid coloboma	−	−	−	−
Hypoplasia of zygomatic complex	+	+	+	+
Microtia	+	−	+	+
Atresia of external ear canal	−	−	+	+
Cleft palate	−	−	+	+
Conductive deafness	+	−	+	+
Choanal atrasia	−	−	−	−
Pre-auricular tags	−	−	+	+
Delayed speech development	+	+	+	+
Antimongoloid stout	+	−	+	+
Ear pinna deformed	−	−	+	+
Micrognathia	+	+	+	−
Partial absence of lower eyelash	−	+	+	+

Facial phenotype	Mild	Mild	Severe	Severe

**Table 2:** Mosaicism of deletions 5q and 3q in the affected members.

Affected members	Karyotype	%
II.2	46,XX	60
	46,XXdel(5q32-33)	30
	46,XXdel(3q23–25)	10
III.3	46,XX	53
	46,XXdel(5q32-33)	47
IV.1	46,XY	45
	46,XYdel(5q32-33)	55
IV.2	46,XX	60
	46,XXdel(5q32-33)	40
